# Syphilis in the Americas: a protocol for a systematic review of syphilis prevalence and incidence in four high-risk groups, 1980–2016

**DOI:** 10.1186/s13643-017-0595-3

**Published:** 2017-10-10

**Authors:** Ken Kitayama, Eddy R. Segura, Jordan E. Lake, Amaya G. Perez-Brumer, Catherine E. Oldenburg, Bethany A. Myers, Paria Pourjavaheri, Chinomnso N. Okorie, Robinson L. Cabello, Jesse L. Clark

**Affiliations:** 1 Department of Medicine, Division of Infectious Diseases, UCLA South American Program in HIV Prevention Research (SAPHIR), David Geffen School of Medicine at UCLA, 10833 Le Conte Ave, CHS 37–121, Los Angeles, CA 90095 USA; 2 Department of Medicine, Division of Infectious Diseases, David Geffen School of Medicine at UCLA, Los Angeles, CA USA; 3 Escuela de Medicina, Universidad Peruana de Ciencias Aplicadas, Lima, Peru; 4 Department of Medicine, Division of Infectious Diseases, McGovern Medical School at the University of Texas Health Science Center at Houston, Houston, TX USA; 5 Columbia University Mailman School of Public Health, New York, NY USA; 6 San Francisco School of Medicine, Francis I. Proctor Foundation for Research in Ophthalmology, Department of Ophthalmology, and Department of Epidemiology and Biostatistics, University of California, San Francisco, CA USA; 7 Louise M. Darling Biomedical Library at UCLA, Los Angeles, CA USA; 8 Department of Biology, San Francisco State University, San Francisco, CA USA; 9 Asociación Civil Vía Libre, Lima, Peru

**Keywords:** Syphilis, Prevalence, Incidence, Men who have sex with men, Transgender women, Sex workers, Incarcerated individuals, Systematic review, Meta-analysis, Protocol

## Abstract

**Background:**

Syphilis infection has recently resurfaced as a significant public health problem. Although there has been a tremendous amount of research on the epidemiology of syphilis, there has been limited work done to synthesize the extensive body of research and systematically estimate patterns of disease within high-risk groups in the Americas. The purpose of this systematic review and meta-analysis is to (1) summarize recent patterns of syphilis infection in North and South America among four high-risk groups (MSM, transgender women, sex workers, and incarcerated individuals) from 1980 to 2016, (2) identify and differentiate regional geographic epidemiologic characteristics, and (3) compare the epidemics of the economically developed countries of North America from the developing countries and public health systems of Latin America and the Caribbean.

**Methods/design:**

Primary studies reporting syphilis prevalence and/or incidence in at least one of the four high-risk groups will be identified from Medline/PubMed, Embase, Lilacs, SciELO, The Cochrane Library, Web of Science, Scopus, ProQuest, CINAHL, Clase, and Periódica, as well as “gray” literature sources (conference abstracts, country reports, etc.). Studies published from 1980 through 2016 will be included. Data will be extracted from studies meeting inclusion and exclusion criteria and a random effects meta-analysis of prevalence and incidence estimates will be conducted. Heterogeneity, risk of bias, and publication bias will be assessed. Pooled prevalence and incidence estimates will be calculated for comparisons based on geographic region, risk factors, and time period.

**Discussion:**

Our systematic review and meta-analysis aims to contribute to an improved understanding of global epidemiologic patterns of syphilis infection in most-at-risk populations. Through systematic classification of the existing literature, and comparison of disease patterns across regional, temporal and socio-behavioral differences, we hope to improve public health surveillance and improve efforts to control the spread of disease across the Americas.

**Systematic review registration:**

PROSPERO CRD42016047306.

**Electronic supplementary material:**

The online version of this article (10.1186/s13643-017-0595-3) contains supplementary material, which is available to authorized users.

## Background

### Global burden of syphilis

Syphilis is a critical public health problem worldwide. Although the incidence of syphilis infection in the USA and Western Europe decreased after the introduction of penicillin in the early 1940s [1, 2], a resurgence in syphilis transmission that began in the 1980s has now gained substantial strength. Beginning in the 2000s, a growing number of isolated syphilis outbreaks were reported in North America, Europe, and Australia, primarily within sexual networks of men who have sex with men (MSM), and often associated with HIV co-infection or co-transmission [3–11]. Following the successful introduction of biomedical approaches to HIV prevention, these sporadic outbreaks have more recently grown in frequency and size to mark a new era of syphilis transmission. In contrast to the relatively more effective control of disease in the USA and Western Europe, endemic rates of syphilis transmission have been stubbornly persistent in the resource-limited public health systems of Latin America, Asia, and Africa [12–21]. Despite the proliferation of epidemiologic reports on syphilis prevalence and incidence, we are not aware of any comprehensive synthesis of the available literature that can (i) summarize recent patterns of syphilis infection and transmission, (ii) identify and differentiate regional epidemiologic characteristics, and (iii) compare the developing syphilis epidemics of the economically developed countries of North America with the endemic patterns of disease observed in Latin America and the Caribbean.

The global epidemiology of syphilis infection varies significantly by geography. A 2015 systematic review based on data from the 2012 Global AIDS Response Progress Reporting (GARPR) system estimated there were approximately 17,721,000 cases of syphilis among men and women 15–49 years of age worldwide [22]. Though country-specific estimates were not provided, the study authors noted differences in syphilis prevalence according to World Bank economic classifications, with prevalence estimates lowest for high-income countries, at 0.2% overall, and ranging from 0.3% to 1.3% in middle- to low-income economies.

### Syphilis in high-risk groups

Risks for syphilis infection among MSM, transgender women (TW), and sex workers (SW) of all genders are disproportionately high across geographic locations, but there have been few comprehensive global estimates of disease prevalence and incidence in these key populations [23–25]. GARPR did not begin collecting behavioral data on syphilis in adults until 2012, with a formal roll out of reporting for all regions initiated in 2013 [26]. As a result, pre-2012 GARPR estimates of syphilis infection among MSM and TW were based on extrapolations from reported data on cases of maternal and congenital syphilis, with the assumption that heterosexual transmission would result in a 1:1 male to female ratio of syphilis prevalence [22]. In Latin America and the Caribbean, a 2013 systematic review of syphilis data in MSM, TW, SW, and clients of SW included 35 studies on syphilis prevalence in MSM, with a prevalence > 4% in three quarters of the estimates included and > 7.5% in half [25]. Five of those studies included TW, with syphilis prevalence ranging from 6.5% in El Salvador to 43.3% in Brazil. The same review identified 49 studies with female SWs, half of them with a syphilis prevalence > 5%, but noted large variations between the populations sampled.

In addition to MSM and SW, the United Nations has also identified incarcerated individuals as a group at high-risk for syphilis and other sexually transmitted infections (STIs), with frequencies of disease two to 10 times higher (and potentially 50 times greater) among prisoners than in the general population worldwide [27]. A 2012 systematic review of incarcerated populations calculated pooled syphilis prevalence estimates of 2.5% in men and 6.1% in women, finding that the incidence of infection among women was 2.7 times greater than in men (95% CI 1.5–4.8) [28].

### Review aims

In order to improve the public health response to the emerging syphilis epidemics in the Americas, detailed knowledge of epidemiologic patterns of disease in most-at-risk populations is needed. A systematic review and analysis of the broad range of existing data will help to establish a framework for understanding the parameters of syphilis transmission across the region and help to define target points for STI control efforts. Comparing patterns of disease in Latin America and the Caribbean with those seen in Canada and the USA will help to illuminate how behavioral, biological, social, and structural characteristics of syphilis transmission differ across geographic, socioeconomic, and epidemiologic contexts, as well as how they may have changed over time. Ultimately, the improved understanding of the global epidemiology of syphilis infection to be provided by our analysis will help to define and allocate future public health surveillance and control efforts. We plan to conduct a systematic review and meta-analysis of data on syphilis infection prevalence and incidence in four high-risk groups (MSM, TW, SW, and incarcerated persons) within Latin America, the Caribbean, and North America (USA and Canada), in published studies and reports from 1980 to 2016.

## Methods and design

This systematic review and meta-analysis protocol was developed according to the Preferred Reporting Items for Systematic Reviews and Meta-Analysis Protocols (PRISMA-P) 2015 statement [29]. The protocol has been published in the PROSPERO International Prospective Register of systematic reviews (http://www.crd.york.ac.uk/PROSPERO), registration number CRD42016047306.

### Criteria for considering studies for review

#### Inclusion criteria

1. Studies reporting primary, quantitative data on the prevalence and/or incidence (cumulative incidence or incidence rates) of active and/or lifetime syphilis infection among at least one high-risk group (MSM, TW, SW, and/or incarcerated persons) who reside in countries and territories within the Americas and who are from any race, ethnicity, socioeconomic status, and/or level of education will be included. For the purpose of this study, “The Americas” refers to all countries designated under this geographical group by the World Health Organization [30].
2. Included studies must describe appropriate criteria used for diagnosis of active syphilis infection, including serologic assays (e.g., rapid plasma reagin (RPR), venereal disease research laboratory test (VDRL), treponema pallidum particle agglutination assay (TPPA), microhemagglutination assay (MHA-TP), treponema pallidum enzyme-linked immunosorbent assay (TP-EIA)), dark-field microscopy, and/or clinical diagnosis based on history and physical exam findings.
3. Articles published in English, Spanish, Portuguese, or French or with sufficient information for data abstraction from an English abstract will be considered for inclusion.

We plan to collect data from articles and reports published from 1 January 1980 through 31 December 2016. This date range selection is based on the fact that in the USA, the highest recorded incidence of syphilis since 1950 was observed in the year 1982 [31], with rates initially declining, but peaking again in 1990–1991, thought to be because of a new surveillance case definition for congenital syphilis [32]. Syphilis infection rates then declined thereafter in association with increased public health efforts to prevent HIV/AIDS and reduce AIDS-related mortality [33, 34] and subsequently followed by a resurgence of syphilis infection among MSM beginning in the late 1990s and early 2000s [35–37].

#### Exclusion criteria

1. Studies or reports that include mixed prevalence or incidence data on syphilis infection as a composite outcome with another STI and that do not specifically report data on syphilis infection alone.
2. Studies or reports that extrapolate syphilis prevalence or incidence data in high-risk groups from general population statistics or congenital syphilis data without direct measurement of the social or behavioral factors used for classification.
3. Publications that report the same data in multiple sources. If the same data is published in more than one article, report, or abstract, the publication reporting the most complete version of the data will be used.
4. Narrative reviews, opinion pieces, letters, or other publications that do not report primary data and/or do not provide sufficient description of the study methodology. Case series and case reports, as well as mathematical modeling studies based on previously reported data, will be excluded.

### Search strategy for identification of relevant studies

The search strategy will be composed of two stages:

Stage 1: bibliographic database review

Ten electronic databases (including MEDLINE/PubMed, Embase, Lilacs, SciELO, The Cochrane Library, Web of Science, Scopus, ProQuest, CINAHL, Clase, and Periódica) will be searched for articles published from January 1, 1980, through December 31, 2016. A list of search terms including “syphilis” or “*Treponema pallidum*” or “*T. pallidum*” or “*pallidum*” in addition to terms for each of the four high-risk groups of interest (MSM, TW, SW, and incarcerated persons) was generated based on strategies from previously published systematic reviews [28, 38–41]. When possible, controlled search terms (MeSH) will be combined with text words. Otherwise, text words alone will be searched. Reference lists of all included articles will be hand-searched to identify additional studies and reports to include in our analysis. For the full search strategy, see Additional file 1.

Stage 2: gray literature search

The Google Scholar search engine will be used to identify “gray” literature (documents not published by commercial publishers such as agency reports, government articles, and academic theses) as previously described [42]. As the Google Scholar search string is limited to 256 characters, a separate search will be conducted for each of the four high-risk groups, and the first 100 results for each of the searches will be reviewed. Conference abstract archives or abstract books will be searched for the following conferences: International AIDS Conference, IAS Conference on HIV Pathogenesis, Treatment and Prevention, Conference on Retroviruses and Opportunistic Infections, IDWeek, and the International Society for Sexually Transmitted Diseases Research. GARPR system country reports, Centers for Disease Control and the Public Health Agency of Canada statistics reports will also be searched. Additionally, we will also poll recognized experts in the field for additional citations or references not identified using other methods.

#### Selecting studies for inclusion

Studies that are found utilizing the search strategies described above will be compiled with their titles and abstracts into EndNote X7 (Thomson Reuters). As EndNote’s ability to accurately remove duplicates has been found to be sub-optimal [43, 44], de-duplication will be performed using Mendeley (Elsevier), which has been found to report fewer false negatives and similar false positives in its de-duplication results when compared to EndNote [45].

The primary author (KK) will apply the inclusion and exclusion criteria to the de-duplicated list of articles, and a separate reviewer (CO) will conduct an independent screen of a random selection of 10% of all titles. Comparison of the two authors’ screen results will be used to calculate an inter-rater reliability score using Cohen’s kappa coefficient [46]. The initial screens will result in the creation of three categories: (1) Included, (2) Excluded, or (3) Pending (when the article initially appears to incompletely fit exclusion and/or inclusion criteria). Titles classified as “Pending” will be examined by a separate author with specialized expertise in the clinical and epidemiologic classification of syphilis infection (JLC) who will make the final determination of inclusion or exclusion. In the full text screen, two reviewers (KK and PP) will independently review the full article texts and apply the inclusion and exclusion criteria listed above. Any disagreements in categorizations within the full-text screen will be resolved by group adjudication. A PRISMA flow diagram outlining the procedures described above will be prepared for transparency and to provide a visual overview of how the final list of articles for data collection was constructed.

#### Data collection

Two reviewers (KK and PP) will independently extract and manage the data for the final set of included studies. A customizable electronic data extraction form will be created using form.io (Dallas, TX) that will be piloted and edited through an iterative process using feedback from the entire panel of authors. Any disagreements in data extraction between KK and PP will be discussed and resolved, with the senior author (JLC) acting as arbiter in instances where disagreements cannot be resolved.

#### Data items

The following data will be extracted:

1. Publication details: year, title, first author, language, type of publication.
2. Study design: study type (cross-sectional, cohort, clinical trial, etc.), sampling method (probability or non-probability sampling and specific type of sampling), recruitment site type (for non-probability sampling).
3. Study participants: city and country where study took place, study time frame, participants’ sex and/or gender, mean/median age and age range, primary (e.g., MSM, TW, SW, incarcerated) and secondary (e.g., mobile app/Internet use to find sex partners, IV drug use) risk-group characteristic(s), number of persons surveyed or studied for per group, HIV serostatus (when available), race, and ethnicity. Diagnostic criteria used to define syphilis infection, syphilis re-infection, and neurosyphilis (if applicable). Number and frequency of syphilis cases, including stage at diagnosis, number and frequency of cases of syphilis re-infection, and number and frequency of cases of neurosyphilis. Reported titers of diagnostic tests (e.g., RPR, VDRL) will be recorded when available.
4. Outcome measures: reported estimates (or, if sufficient raw data is available, estimates calculated by our study team) of the point prevalence, period prevalence, cumulative incidence, and/or incidence rate of active and/or lifetime syphilis in selected areas. Lifetime syphilis will be defined within extracted data as (1) any positive non-treponemal test *with confirmatory treponemal test* or (2) self-reported lifetime history of syphilis infection. Active syphilis will be defined as (1) a four-fold increase in non-treponemal test compared to previous results with positive confirmatory treponemal test or (2) a positive non-treponemal test (e.g., RPR or VDRL) titer of 1:8 or greater with a positive confirmatory treponema-specific test (e.g., TPPA or MHA-TP).

### Critical appraisal of included studies

Quality assessment of the studies will be performed using a nine-item critical appraisal tool to assess the validity and rate the methodological quality of studies reporting prevalence data [47]. Compared to other instruments specifically designed to assess a narrower scope of study designs, this tool is designed to qualify studies of varied designs reporting prevalence data [48]. KK and PP will each independently apply this quality assessment tool to all included studies. Any disagreements will be resolved by consensus discussion and in consultation with the senior author (JLC). The final assessment of risk of bias for all included publications will be presented in a table. Publication bias will be assessed using funnel plots of the study outcome against the sample size, as described by Egger et al. [49]. Furthermore, GRADE guidelines for assessing publication bias will be referred to for additional guidance [50].

### Data synthesis

We will prepare a narrative synthesis and quantitative meta-analysis of the prevalence and incidence of active and lifetime syphilis in the four previously defined high-risk groups in the Americas, with all relevant data presented in tables. Estimates from studies found to be of sufficient quality will be pooled to estimate overall prevalence and incidence of active syphilis infection and overall prevalence and incidence of any syphilis infection, presenting meta-analysis results with forest plots. Data will be displayed graphically as needed to assist in the interpretation of data not amenable to the application of statistical tests for trends.

Because we anticipate elevated levels of heterogeneity in our included studies in terms of study design, outcome measurement, and follow-up (for incidence rates), we will conduct meta-analyses using a DerSimonian and Laird random effects model and will account for heterogeneity of studies by including a parameter for inter-study variation. We will combine the percentages of patients with lifetime and/or active syphilis (as defined above) in each individual study to calculate pooled prevalence estimates along with 95% confidence intervals (CI), using a double arcsine transformation of prevalence.

We will conduct separate subgroup analyses of the data extracted from all studies: (1) between-group analyses of the four high-risk groups (MSM, TW, SW, prisoners) by calculating estimates separately for each group and investigating differences, (2) differences between geographic sub-regions (Latin America and the Caribbean versus North America), (3) changes in syphilis prevalence and incidence over time, as stratified by 10-year intervals, (4) estimates within specific subgroups according to HIV serostatus; and (5) differences in estimates depending on a diagnostic method (self-reported history, serologic testing strategy). Heterogeneity will be assessed with *I* ^2^ and *τ* ^2^ statistics. Sources of heterogeneity between included studies will be examined by stratifying data according to study characteristics. All analyses will be conducted using Stata 14.0 (StataCorp, College Station, TX).

## Discussion

This systematic review and meta-analysis of syphilis in the Americas will compile and analyze the available epidemiologic literature, summarizing the burden of syphilis in high-risk groups in Latin America, the Caribbean, the USA, and Canada. We will collect prevalence and incidence data on active and lifetime syphilis in four high-risk groups (MSM, TW, SW, incarcerated persons), comparing and contrasting geographic regions within the Americas and across time periods from 1980 to 2016. We will also aim to better characterize details and potential nuances of the current epidemiology of syphilis transmission, including the impact on disease incidence of new biomedical prevention technologies like pre-exposure prophylaxis, the use of Internet/mobile applications to find sex partners, and rates of HIV co-infection.

Based on an informal, preliminary review of the existing literature, we expect that there will be tremendous diversity in the prevalence and incidence of syphilis infection between high-risk groups in the USA and Canada as compared with Latin America and the Caribbean. To guide the analysis, we propose the following hypotheses: (1) The prevalence and incidence of syphilis infection in Latin America and the Caribbean will be significantly higher than the prevalence/incidence of infection in the USA and Canada; (2) The majority of syphilis cases reported in the USA and Canada will be identified during primary or secondary syphilis infection (diagnosed through symptomatic illness or network tracing), while the majority of cases in Latin America and the Caribbean will be diagnosed as latent disease (asymptomatic cases identified through serologic testing); and (3) In all regions, there will be a close epidemiologic association between syphilis infection and HIV acquisition and transmission.

Our systematic review and meta-analysis aims to contribute to an improved understanding of global epidemiologic patterns of syphilis infection in most-at-risk populations. Through systematic classification of the existing literature and comparison of disease patterns across regional, temporal, and socio-behavioral differences, we hope to improve public health surveillance and improve efforts to control the spread of disease across the Americas. Understanding the prevalence and incidence of syphilis within and between these high-risk groups will help guide resource allocation and educational efforts, particularly in resource-limited settings in Latin America and the Caribbean.

## Additional file

Additional file 1: Search strategy. (DOCX 106 kb)

## Abbreviations

CI: Confidence interval
GARPR: Global AIDS Response Progress Report
MHA-TP: Microhemagglutination assay
MSM: Men who have sex with men
RPR: Rapid plasma reagin
STI: Sexually transmitted infections
SW: Sex workers
TP-EIA: Treponema pallidum enzyme-linked immunosorbent assay
TPPA: Treponema pallidum particle agglutination assay
TW: Transgender women
VDRL: Venereal disease research laboratory test
