# Supplementary material for: Syphilis in the Americas: a protocol for a systematic review of syphilis prevalence and incidence in four high-risk groups, 1980–2016
Source: Syst Rev. 2017 Oct 10;6:195. doi: 10.1186/s13643-017-0595-3 (PMC5634900; doi:10.1186/s13643-017-0595-3)
Supplement: Additional file 1: — Search strategy. (DOCX 106 kb) [file 13643_2017_595_MOESM1_ESM.docx]

**Supplemental File 1: Search Strategy for PubMed/Medline**

| **Concept** | **Medical Subject Headings (MeSH)** | **Text Words** | **Search strategy** |
| --- | --- | --- | --- |
| **MSM** | Homosexuality[mesh:noexp]  "homosexuality, male"[mesh]  Bisexuality[mesh] | "men who have sex with men"[tw]  MSM[tw]  Gay[tw]  “Bisexual men”[tw] | ((((Homosexuality[mesh:noexp]) OR "Homosexuality, male"[mesh]) OR Bisexuality[mesh]) OR "men who have sex with men"[tw]) OR MSM[tw] OR gay[tw] OR “bisexual men”[tw] |
| **Transwomen** | "transgender persons"[mesh]  "Transsexualism"[mesh] | transwomen[tw]  transgender*[tw]  travesty[tw]  “male to female transgender”[tw]  MTF[tw]  transsexual*[tw]  transvest*[tw]  “cross dresser”[tw]  “cross dressers”[tw]  “cross dressing”[tw]  “cross dressed”[tw]  crossdress*[tw] | (("transgender persons"[mesh] OR "Transsexualism"[mesh])) OR (transwomen[tw] OR transgender*[tw] OR travesty[tw] OR “male to female transgender”[tw] OR MTF[tw] OR transsexual*[tw] OR transvest*[tw] OR “cross dresser”[tw] OR “cross dressers”[tw] OR “cross dressing”[tw] OR “cross dressed”[tw] OR crossdress*[tw]) |
| **Sex Workers** | “sex workers”[mesh] | “sex workers”[tw]  “commercial sex”[tw]  “sex work”[tw]  “transactional sex”[tw]  prostitution[tw]  prostitute[tw]  “exchange sex”[tw] | ("sex workers"[mesh]) OR (“sex workers”[tw] OR “commercial sex”[tw] OR “sex work”[tw] OR “transactional sex”[tw] OR prostitution[tw] OR prostitute[tw] OR “exchange sex”[tw]) |
| **Prisoners** | Prisoners[mesh] | prisoner*[tw]  incarcerated[tw]  imprisoned[tw]  inmate*[tw]  prison*[tw]  jail*[tw]  incarceration[tw]  correctional facility[tw]  correctional facilities[tw]  corrections[tw]  “juvenile detention”[tw] | (prisoners[mesh]) OR (prisoner*[tw] OR incarcerated[tw] OR imprisoned[tw] OR inmate*[tw] OR prison*[tw] OR jail*[tw] OR incarceration[tw] OR “correctional facility” OR “correctional facilities”[tw] OR corrections[tw] OR “juvenile detention”[tw]) |
| **Syphilis** | Syphilis[mesh] | Syphilis[tw]  “treponema pallidum”[tw]  pallidum[tw] | (((syphilis[mesh]) OR "treponema pallidum"[mesh]) OR syphilis[tw]) OR "treponema pallidum"[tw] OR pallidum[tw]) |

Filters: Publication date from 1980/01/01 to 2016/12/31

Search (((((((((Homosexuality[mesh:noexp]) OR "Homosexuality, male"[mesh]) OR Bisexuality[mesh]) OR "men who have sex with men"[tw]) OR MSM[tw] OR gay[tw] OR “bisexual men”[tw])) OR ((("transgender persons"[mesh] OR "Transsexualism"[mesh])) OR (transwomen[tw] OR transgender*[tw] OR travesty[tw] OR “male to female transgender”[tw] OR MTF[tw] OR transsexual*[tw] OR transvest*[tw] OR “cross dresser”[tw] OR “cross dressers”[tw] OR “cross dressing”[tw] OR “cross dressed”[tw] OR crossdress*[tw]))) OR (("sex workers"[mesh]) OR (“sex workers”[tw] OR “commercial sex”[tw] OR “sex work”[tw] OR “transactional sex”[tw] OR prostitution[tw] OR prostitute[tw] OR “exchange sex”[tw]))) OR ((prisoners[mesh]) OR (prisoner*[tw] OR incarcerated[tw] OR imprisoned[tw] OR inmate*[tw] OR prison*[tw] OR jail*[tw] OR incarceration[tw] OR “correctional facility” OR “correctional facilities”[tw] OR corrections[tw] OR “juvenile detention”[tw]))) AND ((((syphilis[mesh]) OR "treponema pallidum"[mesh]) OR syphilis[tw]) OR "treponema pallidum"[tw] OR pallidum[tw])) Filters: Publication date from 1980/01/01 to 2016/12/31
